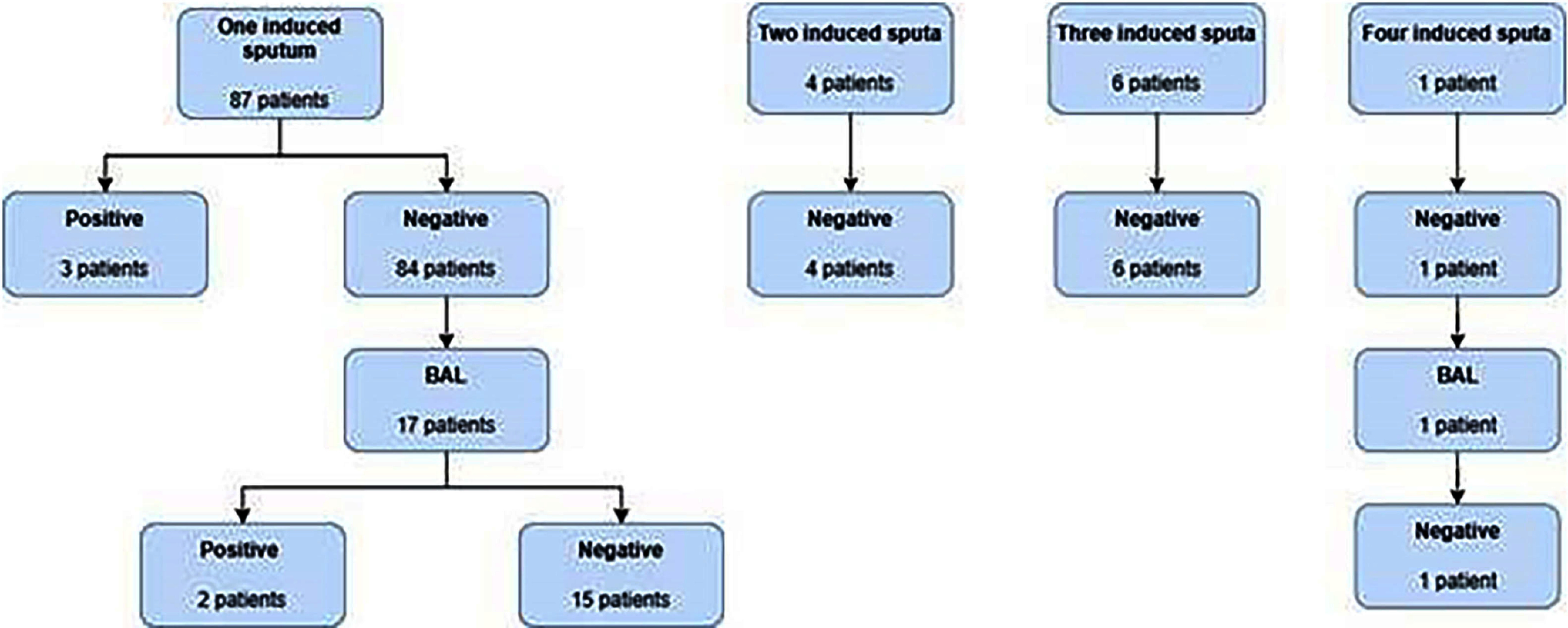# Utility of a Pulmonary TB diagnostic Algorithm to Guide Testing and Airborne Infection Isolation

**DOI:** 10.1017/ash.2025.392

**Published:** 2025-09-24

**Authors:** Qasim Mohiuddin, Susy Hota, Alon Vaisman

**Affiliations:** 1 University Health Network; 2 University Health Network; 3 Infection Prevention and Control, University Health Network

## Abstract

**Background:** Patients with suspected pulmonary TB tuberculosis (PTB) often require scarce airborne isolation rooms; minimizing use depends on clinician understanding of sputum and bronchoscopic test characteristics. Limited knowledge can lead to over-testing and unnecessary isolation days, straining hospital resources. **Objective:** Evaluate the impact of a PTB screening algorithm on reducing unnecessary testing and excess isolation days in patients with low to moderate pre-test probability. **Methods:** The study occurred 2022–2024 at a 1,286-bed tertiary care hospital in Toronto, Ontario (~880 TB cases annually). Inclusion criteria included inpatients placed on airborne isolation for suspected PTB with orders for either ≥3 expectorated sputa, ≥1 induced sputum, bronchoscopy, or combinations thereof. Patients with suspected *Mycobacterium avium* complex were excluded. A positive case is TB PCR or culture positive. Harm is defined as PTB exposure due to premature discontinuation of isolation. The algorithm recommended clinicians to collect a single induced sputum for low/moderate-risk patients with additional testing reserved for high-risk cases. **Results:** A total of 1,152 samples were collected from 747 patients; 513 expectorated sputa (44%), 194 induced sputa (16.8%), 445 bronchoscopies (38.6%). The median isolation duration was 6 days and the turnaround time for results ranged from 3–11 days. The positivity rate was 0.2% for performing expectorated sputum first (1/513), 2.5% for performing induced sputum (3/118) first, and 1.8% for BAL performed first (3/169). When comparing repeated induced sputum testing, all the samples were positive from the first specimen (Figure 2). **Conclusion:** These findings illustrate the real-world implications of using a single induced sputum to rule out PTB in low/moderate pre-testing probability patients, potentially leading to the reduction in airborne isolation days. No added harm via patient exposures was detected with the use of this algorithm.

**Figure:**
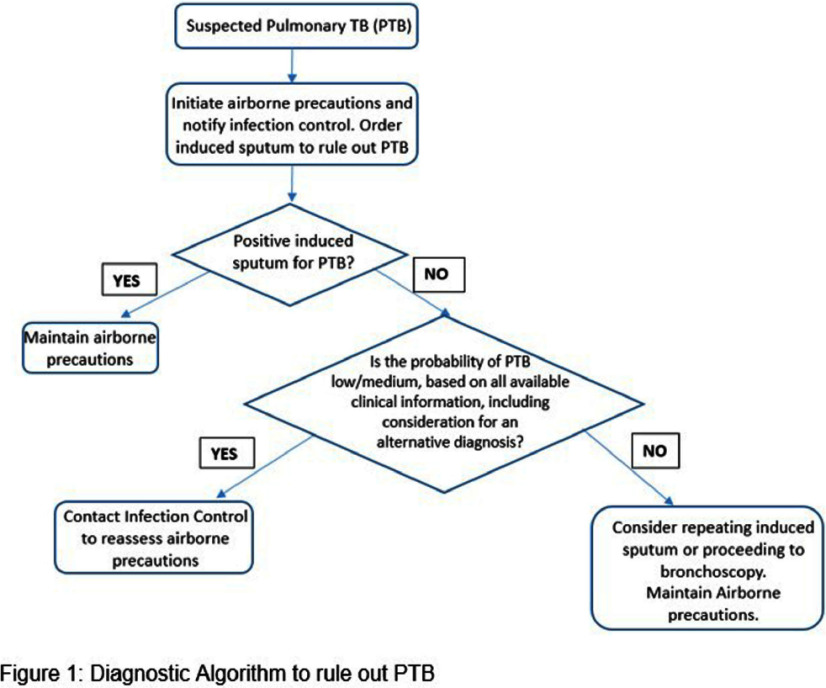

**Figure:**